# Association between preoperative frailty and surgical Apgar score in abdominal cancer surgery: a secondary analysis of a prospective observational study

**DOI:** 10.1186/s40981-024-00687-3

**Published:** 2024-01-13

**Authors:** Sayaka Hirai, Mitsuru Ida, Yuki Kinugasa, Masahiko Kawaguchi

**Affiliations:** 1 Department of Anaesthesiology, Nara Medical University, Kashihara, Japan; 2 Department of Medical Technical Center, Nara Medical University Hospital, Kashihara, Japan

**Keywords:** Apgar score, frailty, neoplasms, surgery

## Abstract

**Introduction:**

The surgical Apgar score is useful for predicting postoperative morbidity and mortality. However, its applicability in frail patients with minimal hemodynamic variation remains unknown. This study aimed to investigate the association between frailty and surgical Apgar score.

**Methods:**

This secondary analysis included 210 patients ≥ 65 years of age undergoing elective major abdominal surgery for cancer. Frailty was assessed using the Fried Frailty Phenotype Questionnaire and defined as a total score of ≥ 3. The surgical Apgar score (range, 0−10; including mean blood pressure, heart rate, and blood loss volume) was compared between patients with or without frailty using the Mann–Whitney U test. Postoperative severe complications and length of postoperative stay were compared between patients with surgical Apgar scores ≤ 7 and > 7.

**Results:**

Among the included patients, 45 were classified as frail. The median [1st quartile, 3rd quartile] surgical Apgar scores in patients with and without frailty were 7.0 [7.0, 8.0] and 8.0 [7.0, 8.0], respectively (P = 0.03). Patients with surgical Apgar score ≤7 had a higher incidence of serious postoperative complications (P = 0.03) and longer hospital stays (P < 0.001) compared with patients with surgical Apgar score >7.

**Conclusion:**

Frail patients have lower SAS, and patients with lower SAS have higher postoperative complication rates and longer hospital stays in patients who underwent cancer surgery.

**Supplementary Information:**

The online version contains supplementary material available at 10.1186/s40981-024-00687-3.

## Background

Frailty is a medical condition characterized by decreased physiological reserve. Recent studies have found that frail patients have a reduced ability to cope with stress, including surgery, which has a strong correlation with postoperative morbidity and mortality in older patients [1–3]. The exact mechanism associated with increased mortality in frail patients is yet to be fully elucidated; however, the involvement of decreased sympathetic reserve, manifested as lesser hemodynamic variation, has been suggested [4].

The surgical Apgar score (SAS) was developed in 2007 to identify patients immediately after surgery who are at a higher risk of experiencing major complications or death within 30 days post-surgery [5]. This novel risk index that integrates three intraoperative parameters (mean blood pressure, heart rate, and blood loss volume) is suitable for routine clinical use. While validation studies across various surgical fields have been published, the presence of frailty has not been taken into consideration in these studies [6–9]. Therefore, limited evidence is available regarding the effects of frailty, with its associated lesser hemodynamic variation, on the SAS, which serves as a reflection of surgical invasion and stress.

We aimed to investigate the potential association between preoperative frailty and the SAS following abdominal cancer surgery. Additionally, the impact of a lower SAS on postoperative complications and hospital stay duration was also assessed.

## Methods

### Ethical approval

This study is a secondary analysis of a prospective observational study, which focused on the effects of 3-month postoperative recovery as measured by the quality of recovery-15 in hospital on disability-free survival. This study was approved by the Nara Medical University Institutional Review Board (Kashihara, Nara, Japan; Chairperson, Prof. M Yoshizumi; approval number: 2975; 28 April 2021). The statistical protocol of this secondary analysis was approved on 17 August 2023; Kashihara, Nara, Japan; Chairperson, Prof. M Yoshizumi; approval number: 2975).

### Inclusion and exclusion criteria

Our initial study, which focused on the effects of postoperative recovery as measured by the quality of recovery-15 in hospital on disability-free survival three months later, included a total of 230 patients aged 65 years or older who underwent elective major abdominal surgery with a cancer diagnosis [10]. Among them, patients without atrial fibrillation and cardiac pacemakers were included in this study.

### Data collection

We collected various preoperative patient characteristic data, including co-morbidities, daily medications, and frailty at the perioperative management center where patients underwent medical interviews and were scheduled for surgery. Frailty was assessed using the Fried Frailty Phenotype Questionnaire, including five domains (fatigue, resistance, ambulation, inactivity, and loss of weight) with the total score ranging from 0 to 5 points [11]. Patients with a total score ≥ 3 were classified as frailty patients [11]. In terms of intraoperative data, we collected information on anesthetics used, total administered dose of ephedrine and phenylephrine, total fluid volume, surgical field, postoperative analgesia, surgical duration, and SAS. The SAS, with a total score of 0 (bad) to 10 (excellent), was calculated based on the following three parameters: lowest mean blood pressure (0–3), lowest heart rate (0–4), and blood loss volume (0–4) [5].

### Anesthetic management

Daily oral medications used by the patients were continued except for angiotensin receptor blockers and angiotensin-converting enzyme inhibitors. No pre-surgery medication was administered on the day of surgery. Patients were allowed to have clear water orally up to two hours before entering the operating room. Intraoperative management, including the insertion of an arterial catheter, fluid therapy, and choice of cardiovascular agents, was determined by the attending anesthesiologist. Mean arterial blood pressure values were recorded at 2.5-minute intervals (when blood pressure was measured using oscillometry) or at 1-minute intervals (when an arterial catheter was used).

### Outcomes

The primary outcome of this study was the SAS. Secondary outcomes were postoperative severe complications, defined as a Clavien–Dindo classification ≥ 3 [12], and length of postoperative stay.

### Statistical analysis

Continuous data are presented as median [1st quartile, 3rd quartile], and categorical variables are presented as number (%). Univariate analysis was performed using the Mann–Whitney U test or Fisher's exact test as appropriate, to compare the two groups (robust vs. frailty). To assess the effect of SAS on postoperative severe complications and length of postoperative hospital stays, a cut-off value of SAS 7 was determined because of median SAS scores in patients with and without frailty were 7 and 8. Subsequently, the secondary outcomes were compared between patients with SAS ≤ 7 or >7.

Since this study involved a secondary analysis, sample size calculation was not performed. However, as an alternative, we performed a post hoc power analysis using G*power version 3.1 (Faul, Erdfelder, Lang, & Buchner, 2007) with a type I error of 0.05 and effect size of 0.5 (large effect size). With these parameters and the existing number of patients (robust = 165 and frailty = 45), the power was determined to be 0.82 to detect a significant difference. IBM SPSS Statistics (version 25.0; IBM Corp., Armonk, NY) was used to analyze all data, and p-values < 0.05 were considered statistically significant.

One post hoc analysis using nonlinear restricted cubic splines in the regression model was performed to confirm the nonlinearity of SAS for secondary outcomes.

## Results

**Fig. 1:**
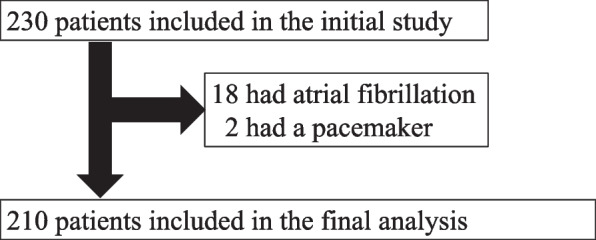
Patient flowchart

**Fig. 2:**
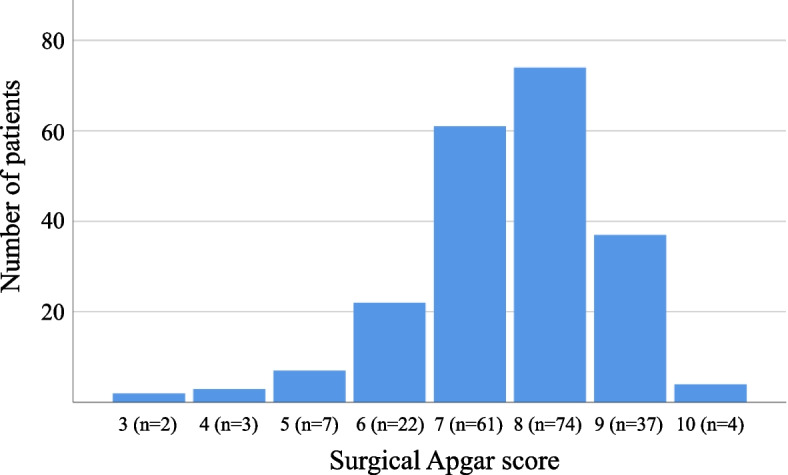
Distribution of surgical Apgar score

Out of the initial 230 patients, a total of 210 patients were included in this study (Fig. 1). Among them, 165 patients were classified as robust and 45 as frail. There were no statistically significant differences in preoperative characteristics between the two groups, except for sex (P = 0.01), serum albumin (P < 0.001), and blood loss volume (p=0.01) (Table 1). The distribution of SAS is shown in Fig. 2 and Supplemental Table 1. The median [1st quartile, 3rd quartile] values of SAS were 7.0 [7.0, 8.0] and 8.0 [7.0, 8.0] in patients with or without frailty, respectively, which was statistically significant (*P* = 0.03) (Table 1).

**Table 1:** Preoperative and intraoperative characteristics

	Total (*n*=210)	Robust (*n*=165)	Frailty (*n*=45)	P-value
Age (yr)	73 [69, 77]	73 [69, 77]	73 [70, 78]	0.38
Male	147 (70.0)	123 (74.5)	24 (53.3)	0.01
Body mass index (kg/m^2^)	22.9 [20.9, 24.8]	23.1 [20.9, 24.7]	21.9 [21.0, 25.9]	0.80
ASA-PS				
1	9 (4.3)	7 (4.2)	2 (4.4)	0.73
2	170 (81.0)	135 (81.8)	35 (77.8)	
3	31 (14.8)	23 (13.9)	8 (17.8)	
Comorbidity				
Symptomatic cerebral vascular disease	10 (4.8)	8 (4.8)	2 (4.4)	> 0.99
Hypertension	117 (55.7)	90 (54.5)	27 (60.0)	0.61
Ischemic heart disease	12 (5.7)	11 (6.7)	1 (2.2)	0.46
Peripheral arterial disease	1 (0.5)	1 (0.6)	0 (0.0)	> 0.99
Diabetes	53 (25.2)	40 (24.2)	13 (28.9)	0.56
Medication				
β-blocker	11 (5.2)	6 (3.6)	5 (11.1)	0.06
Steroid	3 (1.4)	3 (1.8)	0 (0.0)	> 0.99
Statin	57 (27.1)	42 (25.5)	15 (33.3)	0.34
Laboratory data				
Serum albumin (g/dL)	4.2 [4.0, 4.4]	4.3 [4.1, 4.5]	4.1 [3.7, 4.2]	<0.001
Serum creatinine (mg/dL)	0.80 [0.67, 0.99]	0.81 [0.68, 1.00]	0.77 [0.65, 0.93]	0.18
Intraoperative covariate				
Anesthetic agents				
Inhalation agents	203 (96.7)	160 (97.0)	43 (95.6)	0.64
Intravenous agents	7 (3.3)	5 (3.0)	2 (4.4)	
Surgical field				0.13
General	155 (73.8)	121 (73.3)	34 (75.6)	
Urologic	49 (23.3)	41 (24.8)	8 (17.8)	
Gynecologic	6 (2.9)	3 (1.8)	3 (6.7)	
Insertion of arterial catheter	167 (79.5)	129 (78.2)	38 (84.4)	0.41
Total fluid volume (mL)	2300 [1750, 3300]	2250 [1745, 3262]	2500 [1912, 4087]	0.21
Ephedrine (mg)	14.0 [8.0, 24.0]	12.0 [6.0, 24.5]	16.0 [8.0, 21.0]	0.71
Phenylephrine (mg)	0.10 [0.0, 0.75]	0.10 [0.0, 0.56]	0.30 [0.0, 0.85]	0.26
Postoperative analgesia				0.06
None	2 (0.9)	1 (0.6)	1 (2.2)	
PCEA	95 (45.2)	75 (45.5)	20 (44.4)	
IV-PCA	113 (53.8)	89 (53.9)	24 (53.3)	
Surgical duration (min)	298 [217, 375]	288 [216, 375]	323 [219, 387]	0.63
Blood loss volume (mL)	66 [17, 250]	51 [15, 225]	165 [65, 290]	0.01
Surgical Apgar score	8.0 [7.0, 8.0]	8.0 [7.0, 8.0]	7.0 [7.0, 8.0]	0.03

Median [interquartile range] or number (%) *ASA-PS* American Society of Anesthesiologists-physical status: *PCEA* patient-controlled epidural analgesia: *IV-PCA* intravenous patient-controlled analgesia

Patients with SAS ≤ 7 had a higher rate of serious postoperative complications (11.6% vs. 3.5%, P = 0.03) and a longer duration of hospital stay (10.0 vs. 9.0 days, P < 0.001) compared to patients with SAS >7 (Table 2).

**Table 2:** Postoperative outcomes

	Surgical Apgar score >7 (*n*=115)	Surgical Apgar score ≤ 7 (*n*=95)	*P*-value
Clavien-Dindo classification ≥ 3	4 (3.5)	11 (11.6)	0.03
Length of hospital stay (days)	9.0 [7.0, 10.0]	10.0 [8.0, 15.0]	<0.001

Median [interquartile range] or number (%)

Moreover, a post hoc analysis using nonlinear restricted cubic splines in the regression model demonstrated the nonlinearity of SAS for secondary outcomes (Supplemental Figure 1).

## Discussion

This secondary analysis involving 210 patients undergoing abdominal cancer surgery, revealed that frail patients had a lower SAS. Furthermore, patients with a SAS ≤ 7 exhibited a higher rate of postoperative severe complications and a longer duration of hospital stay compared to those with a SAS > 7.

Although frail patients had lower SAS, a significant difference was observed only for blood loss. Intraoperative blood loss caused by surgical trauma is difficult to control by an anesthesiologist. Although the total dose of cardiovascular agents and fluid volume were not statistically different between the two groups, heart rate and blood pressure were likely adjusted using cardiovascular agents. The exact mechanism between frailty and large blood loss volume remains unclear; however, frail patients exposed to a higher inflammatory status may have increased tissue vulnerability [13].

As expected, patients with lower SAS had worse postoperative outcomes. Some previous studies have adopted different cut-off values for postoperative risk stratification [7, 14–16]. Although our study used a cut-off value of 7, a sensitivity analysis using a cut-off value of 6 based on the study by Gawande et al. [5] also confirmed the impact of SAS on postoperative outcomes (Supplemental Table 2). However, a post hoc analysis demonstrated the nonlinearity of SAS for secondary outcomes (Supplemental Figure 1). This suggested that converting continuous variables to categorical variables might not be required [17].

This study had several limitations. First, the use of different assessment tools for frailty may have affected the results. Various instruments for assessing frailty are currently available, and some instruments require measuring gait speed. In contrast, the Fried Frailty Phenotype Questionnaire used in this study is a measurement tool that assesses frailty only through a questionnaire survey. Second, frailty may increase with the progression of the cancer. However, since metastatic or recurrent cancers do not have a stage classification, we could not include the stage of the cancer in this analysis. Third, the generalizability of the findings is limited due to the study being conducted at a single center and including only patients undergoing elective surgery. Forth, we could not determine the causal relationship between frailty and lower SAS. Finally, univariate analysis was performed to assess the association between frailty and SAS; however, no previous study has evaluated factors associated with SAS. Future studies should investigate the factors associated with SAS that may contribute to worsening postoperative outcomes.

## Conclusions

This study demonstrated that frail patients have lower SAS, and patients with lower SAS have higher postoperative complication rates and longer hospital stays in patients who underwent cancer surgery.

### Supplementary Information

**Additional file 1.**

**Additional file 2.**

## Data Availability

The data pertaining to this study are available as a spreadsheet file upon reasonable request.

## Abbreviations

SAS: surgical Apgar score
