# Supplementary material for: Association between preoperative frailty and surgical Apgar score in abdominal cancer surgery: a secondary analysis of a prospective observational study
Source: JA Clin Rep. 2024 Jan 13;10:2. doi: 10.1186/s40981-024-00687-3 (PMC10787715; doi:10.1186/s40981-024-00687-3)
Supplement: Supplementary file 1 — Additional file 1. [file 40981_2024_687_MOESM1_ESM.pptx]

## Slide 1
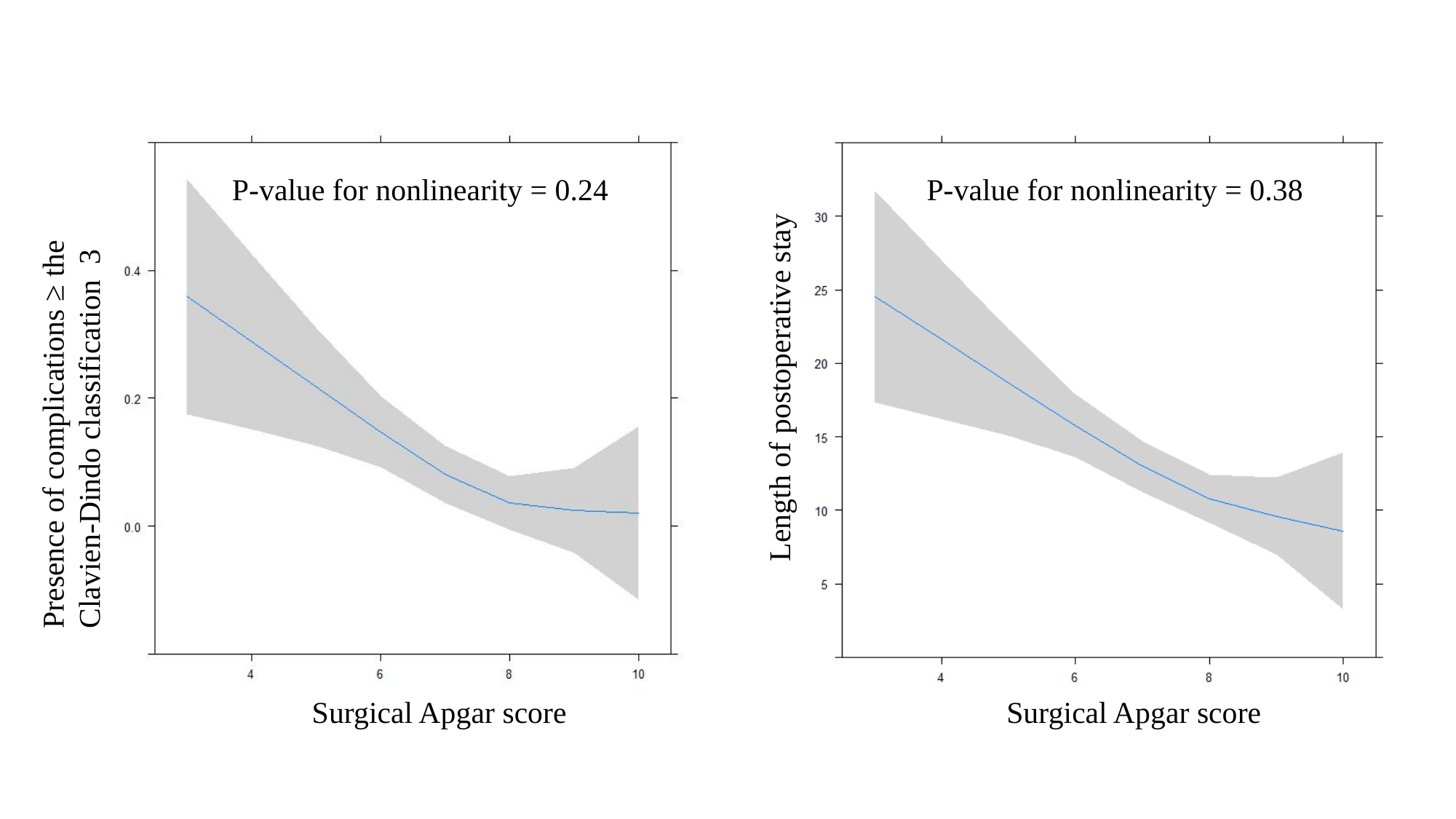

P-value for nonlinearity = 0.24
P-value for nonlinearity = 0.38
Presence of complications ≥ the Clavien-Dindo classification 3
Length of postoperative stay
Surgical Apgar score
Surgical Apgar score
