# Supplementary material for: Association between preoperative frailty and surgical Apgar score in abdominal cancer surgery: a secondary analysis of a prospective observational study
Source: JA Clin Rep. 2024 Jan 13;10:2. doi: 10.1186/s40981-024-00687-3 (PMC10787715; doi:10.1186/s40981-024-00687-3)
Supplement: Supplementary file 2 — Additional file 2. [file 40981_2024_687_MOESM2_ESM.docx]

Supplementary Table 1. The distribution of surgical Apgar score component

|  | Total (n=210) | Robust (n=165) | Frailty (n=45) | P-value |
| --- | --- | --- | --- | --- |
| Lowest mean blood pressure (mmHg) | | |  |  |
| 0 (< 40) | 7 (3.3) | 5 (3.0) | 2 (4.4) | 0.15 |
| 1 (40 – 54) | 114 (54.2) | 85 (51.5) | 29 (64.4) |  |
| 2 (55 – 69) | 83 (39.5) | 71 (43.0) | 12 (26.6) |  |
| 3 (≥ 70) | 6 (2.8) | 4 (2.4) | 2 (4.4) |  |
| Median score | 1.0 [1.0, 2.0] | 1.0 [1.0, 2.0] | 1.0 [1.0, 2.0] | 0.11 |
| Lowest heart rate (beats/min) | |  |  |  |
| 0 (> 85) | 0 (0.0) | 0 (0.0) | 0 (0.0) | 0.68 |
| 1 (76 – 85) | 1 (0.4) | 1 (0.6) | 0 (0.0) |  |
| 2 (66 – 75) | 13 (6.1) | 9 (5.4) | 4 (8.8) |  |
| 3 (56 – 65) | 55 (26.1) | 45 (27.3) | 10 (22.2) |  |
| 4 (≤ 55) | 141 (67.1) | 110 (66.7) | 31 (68.9) |  |
| Median score | 4.0 [3.0, 4.0] | 4.0 [3.0, 4.0] | 4.0 [3.0, 4.0] | 0.88 |
| Blood loss (mL) |  |  |  |  |
| 0 (> 1,000) | 7 (3.3) | 5 (3.0) | 2 (4.4) | 0.004 |
| 1 (601 –1,000) | 7 (3.3) | 7 (4.2) | 0 (0.0) |  |
| 2 (101 – 600) | 72 (34.2) | 46 (27.9) | 26 (57.7) |  |
| 3 (≤ 100) | 124 (59.0) | 107 (64.8) | 17 (37.7) |  |
| Median score | 3.0 [2.0, 3.0] | 3.0 [2.0, 3.0] | 2.0 [2.0, 3.0] | 0.004 |

Median [interquartile range] or number (%)

Supplementary Table 2. Postoperative outcomes among patients with Surgical Apgar score > 6 or ≤ 6

|  | Surgical Apgar score > 6 (n=176) | Surgical Apgar score ≤ 6 (n=34) | P-value |
| --- | --- | --- | --- |
| Clavien-Dindo classification ≥ 3 | 8 (4.5) | 7 (20.5) | <0.001 |
| Length of hospital stay | 9.0 [7.0, 11.0] | 12.0 [8.0, 22.0] | <0.001 |

Median [interquartile range] or number (%)
